# Integrative transcriptomic and gene co-expression network analysis of host responses upon *Verticillium dahliae* infection in *Gossypium hirsutum*

**DOI:** 10.1038/s41598-021-99063-9

**Published:** 2021-10-18

**Authors:** Guoli Zhang, Zengqiang Zhao, Panpan Ma, Yanying Qu, Guoqing Sun, Quanjia Chen

**Affiliations:** 1 College of Agriculture, Xinjiang Agricultural University, 311 Nongda East Road, Urumqi, 830052 China; 2 Biotechnology Research Institute, Xinjiang Academy of Agricultural and Reclamation, 221 Wuyi Highway, Shihezi, Xinjiang 832000 China; 3 Biotechnology Research Institute , Chinese Academy of Agricultural Sciences, Beijing, 100081 China

**Keywords:** Plant sciences, Pathogens

## Abstract

Worldwide, Verticillium wilt is among the major harmful diseases in cotton production, causing substantial reduction in yields. While this disease has been extensively researched at the molecular level of the pathogen, the molecular basis of *V. dahliae* host response association is yet to be thoroughly investigated. In this study, RNA-seq analysis was carried out on *V. dahliae* infected two *Gossypium hirsutum* L. cultivars, Xinluzao-36 (susceptible) and Zhongzhimian-2 (disease resistant) for 0 h, 24 h, 72 h and 120 h time intervals. Statistical analysis revealed that *V. dahliae* infection elicited differentially expressed gene responses in the two cotton varieties, but more intensely in the susceptible cultivar than in the resistant cultivars. Data analysis revealed 4241 differentially expressed genes (DEGs) in the LT variety across the three treatment timepoints whereas 7657 in differentially expressed genes (DEGs) in the Vd592 variety across the three treatment timepoints. Six genes were randomly selected for qPCR validation of the RNA-Seq data. Numerous genes encompassed in disease resistance and defense mechanisms were identified. Further, RNA-Seq dataset was utilized in construction of the weighted gene co-expression network and 11 hub genes were identified, that encode for different proteins associated with lignin and immune response, Auxin response factor, cell wall and vascular development, microtubule, Ascorbate transporter, Serine/threonine kinase and Immunity and drought were identified. This significant research will aid in advancing crucial knowledge on virus-host interactions and identify key genes intricate in *G. hirsutum* L. resistance to *V. dahliae* infection.

## Introduction

Cotton (*Gossypium* spp.) is an economically vital plant which is grown worldwide. The cotton verticillium wilt disease is a severe vascular disease which is characterized by an adverse effect on the yield of cotton and fiber quality^1^ with the etiological agent being the soil-borne fungus *Verticillium dahliae* Kleb. Wilting, yellowing, defoliation and finally death of cotton plants are caused by this fungus^2^, and it is difficult to monitor the pathogens as a result of their long-term survival in the soil as microsclerotia and their wide variety of hosts^3^.

*Verticillium dahliae* microsclerotia overcome soil mycostatic action and germinate to the root in existence of root exudates^4^. The hyphae invades host in order to establish a penetration link to the root epidermal cells through the development of an infections structure, called hyphopodium^5^. They penetrate the Xylem vessels and clog it, leading to a curl, necrosis, defoliation and discoloration of the leaves of the vascular tissue^6^. During its growth cycle, cotton is incessantly exposed to *V. dahliae* infection. Huge portions of China’s cotton fields suffer from *V. dahliae* infection, and can reduce yields up to 50%, even wiping the cultivation entirely.

To combat *V. dahliae* challenge, resistance in cotton has developed various levels of defensive mechanisms during long coexistence and arm race, including tissue structure, physiological and biochemical resistance^7,8^. In the recent past, significant advancement has been achieved in the use of genomics, transcriptomics and proteomics with aim of gaining insight on the genetic mechanism behind cotton resistance to *V. dahliae*, and a variety of *V. dahliae* resistance related genes have been revealed^3,9–13^. However, given the common evolving interaction between cotton and *V. dahliae*, it is critical to investigate the molecular pathways that determine *V. dahliae* pathogenicity. Due to the completion of genome sequencing and the advancement of bioinformatics methods, genomic and transcriptomic sequence knowledge of *V. dahliae* offers us with an ability to better understand its pathogenicity. The transcriptomic analysis of *V. dahliae* during microsclerotia establishment and initial infection phase have provided a glimpse of the genes essential for *V. dahliae* growth, microsclerotia formation, and infection^14–16^. For example, VdPKAC1, VMK1, VdMsb, VdGARP1, VDH1, Vayg1 and VGB were discovered to be intricate in the development of microsclerotia and the pathogenic phase of *V. dahliae*^17,18^; VdNEP, VdpevD1, VdNLP1 and VdNLP2 encode for effector proteins are linked in pathogenic responses^19–22^; VdFTF1, Vta2 and VdSge1 encode for transcriptional factors that regulate pathogenic genes^23–25^. Nonetheless, owing to the intricacy of *V. dahliae* pathogenic associated molecular mechanisms, the functions of these genes in the interaction between *V. dahliae* and cotton are yet to be fully understood. In the present study, we utilized *V. dahliae* fungus in infecting two *Gossypium hirsutum* L. cultivars, Xinluzao-36 (susceptible) and Zhongzhimian-2 (disease resistant) for 0 h, 24 h, 72 h and 120 h time intervals, so as to determine the molecular basis of *V. dahliae* host response mechanisms.

## Methodology

### *V. dahliae* culture

A defoliating *V. dahliae* Vd592 strain isolate which was acquired from Shihezi University, was cultured on potato dextrose agar (PDA) for a period of 7 days at a temperature of 25 °C. The cultured isolates were incubated in Czapek liquid medium for a period of 5 days at a temperature of 25 °C, so as to get the conidia. Using distilled water, the obtained spores were diluted to almost 1 × 10^6^ spores per milliliter prior to inoculation.

### Plant material and inoculation procedure

Xinluzao-36 (susceptible) and Zhongzhimian-2 (disease resistant) cotton cultivars that were used in this study were collected from Shihezi University, a kind gift from Professor Li Guoying. A syringe needle was used for acupuncture, and thereafter 10 mL of spore suspension was injected into the surrounding soil at the acupuncture site. Sampling was conducted at 0 h, 24 h, 72 h and 120 h time intervals, and afterwards subjected to transcriptome sequencing.

### RNA extraction and sequencing

As per the manufacturer’s guidelines, total RNA was extracted from the study samples using Trizol Reagent (Invitrogen, Life Technologies, Carlsbad, CA, USA). Using Oligo (dT) magnetic beads, purified poly (A) + mRNA was isolated from total RNA. By applying a fragmentation buffer to the mRNA, it was sheared into short fragments. Utilizing random primers and SuperScript II, first-strand cDNA was produced from the short poly (A) + mRNA fragments. To yield a second-strand cDNA, RNaseH, DNA polymerase I, Buffer and dNTPs were added. End pairing of the double-stranded cDNA was achieved through addition of T4 DNA polymerase, Klenow Enzyme, and T4 polynucleotide kinase enzyme. Subsequently, DNA ligase was used to ligate the sequencing adapters to the fragments, and thereafter a single ‘A' base attachment with Klenow 3–5′ exo-polymerase. The cDNA fragments were isolated on an agarose gel and used as sequencing templates for high-throughput sequencing. Sequencing was done on Illumina HiSeq 2000 platform.

### Data and differentially expressed gene analysis

Quality assurance checks were performed on the sequenced raw reads. Poor quality reads were filtered out and adapter sequences together with reads containing ambiguous bases ‘N’ and ones that had more than 20% Q < 30 bases were discarded. Following Meyer et al., all sequences of less than 60 bp were also discarded^26^. Using TopHat version 2.01^27^, the obtained clean reads were mapped to the *G. hirsutum* (AD1) reference genome^28^. The Cufflinks software (version 2.2.1) was utilized in measuring the abundance transcripts and differential gene expression^27^. Comparing gene expression levels amongst the two libraries was achieved through the relative transcript level of individually expressed gene was achieved through the calculation and normalization to the reads per kilobase of the exon model per million mapped reads (RPKM)^29^. Detection of the considerable variations and differences in gene expression patterns was done through the Chi-squared test that was incorporated in the IDEG6 software^30^. P-value threshold was defined through false discovery rate (FDR) so as to account for the multiple significance tests. In the present study, an FDR threshold ≤ 0.01 and Fold change ≥ 2 were accepted in determining the significantly expressed gene value differences.

In performing functional annotation of the obtained DEGs, Basic Local Alignment Search Tool (BLAST) alignment was executed via search in the Clusters of Orthologous Groups (COG)^31^, SwissProt, Kyoto Encyclopedia of Genes and Genomes (KEGG)^32,33^ and Non-redundant (Nr) protein databases with the E-value set at ≤ 1e−5. Significant matches were carefully chosen in annotation of the DEGs. Blast2go software version 5.2.5 was utilized in annotating the DEGs’ major Gene Ontology (GO) groups, including molecular functions, biological processes, and cellular components and the E-value ≤ 1e−5^34^.

### Real-time quantitative PCR (RT-qPCR) analysis of the DEGs

Sequencing libraries were prepared from the total RNA, as described above. For RT-qPCR analysis, 6 randomly selected DEGs and cotton gene as the control were chosen, and primer design was done using DNAMAN 6.0 software package. RT-qPCR was done in an ABI 7900HT Real Time PCR System (Applied Biosystems, Life Technologies, Carlsbad, CA, USA) and a SYBR Premix Ex Taq (Tli RNaseH Plus), ROX plus (Takara Bio Inc., Shiga, Japan). The utilized thermocycling settings were: denaturation for 30 s at a temperature of 95 °C, followed by 40 cycles at a temperature of 95 °C for 5 s, and 60 °C for 30 s. The 2 − ΔΔCT procedure was used to normalize and calibrate the relative expression levels^35^. For each of the chosen genes, tests were performed in triplicates for all the biological and technical groups.

### Statistical analysis and graphic presentation

R statistical package version 3.6.2 was utilized in experimental data analysis^36^. Prcomp software package was employed in Principal components analysis (PCA) analysis as well as graphic demonstration was executed via the scatterplot3d package. The boxplot package was used in the graphic demonstration of expression level distribution. The hierarchical clustering package was utilized in clustal analysis whereas the cluster tree graphic presentation and heatmap were achieved by means of the ggplots package. The WGCNA package version 1.70-3 was applied in preparing the graphical outlook of the network/module construction^37^.

### Statement

The two cotton cultivars that were used in this study were collected from Shihezi University. Its growth, harvest and use in this manuscript complies with relevant institutional, national, and international guidelines and legislation.

## Results

**Figure 1:**
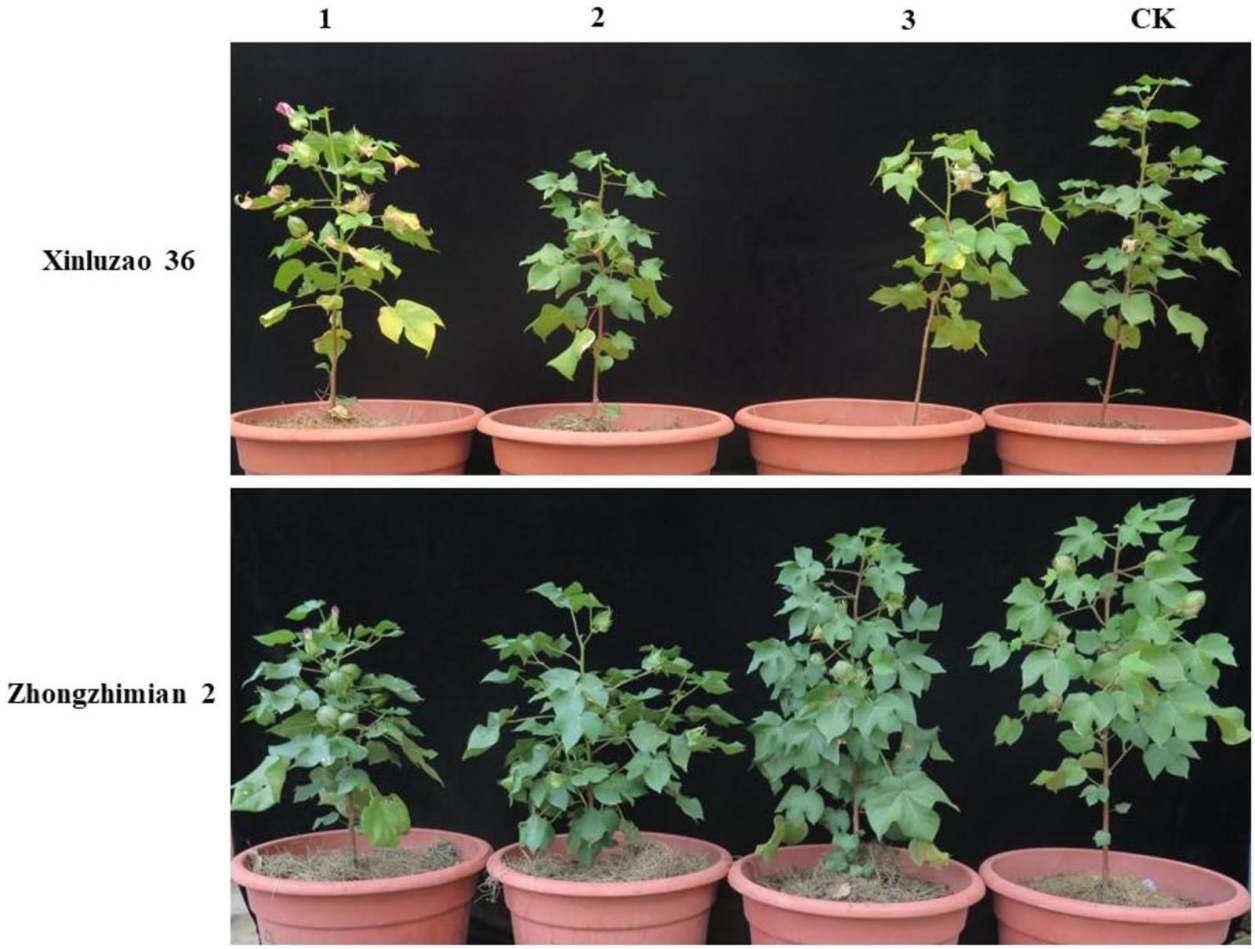
Morphologic and physiologic features of Xinluzao-36 and Zhongzhimian-2 subsequent to *V. dahliae* fungus infection.

The two cotton genotypes morphologically are identical, However, Xinluzhao-36 cultivar exhibited significant variations when exposed to *V. dahliae* fungus infection. This was exhibited by the leaves yellowing and starting to dry up from the edges. Further, it was observed that the Zhongzhimian-2 variety had a large biomass and yellowing of leaves compared to the Xinluzhao-36 cultivar (Fig. 1).

### Sequencing and transcript identification

In this study, two cotton cultivars; Xinluzao-36 (susceptible) and Zhongzhimian-2 (disease resistant) exposed to *V. dahliae* fungus infection were utilized in RNA-Sequencing (Table 1). These two *V. dahliae* strains differ in their pathogenicity, in that the LT strain is a weak pathogenic defoliating isolate, whereas the Vd592 strain is a strong pathogenic defoliating isolate.

**Table 1:** Strains and disease index table.

Strains	Source	Disease index	
		Xinluzao 36 (susceptible variety)	Zhongzhimian 2 (resistant variety)
LT	Luntai, Xinjiang	21.52	8.91
Vd592	Xinjiang	85.43	18.72

The root tissues of the two accessions were considered at three different treatment periods; the samples being labeled as S2-24 h, S2-72 h and S2-120 h and S3-24 h, S3-72 h, S3-120 h for the Xinluzao-36 cotton variant whereas T2-24 h, T2-72 h, T2-120 h and T3-24 h, T3-72 h, T3-120 h for the Zhongzhimian 2 cotton variant (Supp File 1). All the samples were sequenced in triplicates. The two upland cotton accessions were categorized as either *V. dahliae* susceptible variety (S2 and S3) or *V. dahliae* resistant variety (T2 and T3). CK group represented the control plants that grew in normal optimum conditions. To examine the transcriptomic contrasts during *V. dahliae* fungus infection in the treated groups, total RNA was extracted from the root of the two varieties in three different treatment periods.

A total of 1106 million raw reads were obtained from 32 cDNA libraries, and 1097 million (99.38%) clean reads were collected following data quality trimming processes (Supp File 1). For each library the proportion of the mapped data was between 82.97% and 90.01%. In addition, the clean reads were mapped to *G. hirsutum* (AD1) reference genome in which 84.36 to 89.83 percent of the 32 samples obtained, producing 77.4 to 82.3% reads that uniquely mapped to the reference genome.

### Identification and verification of differentially expressed genes (DEGs)

**Figure 2:**
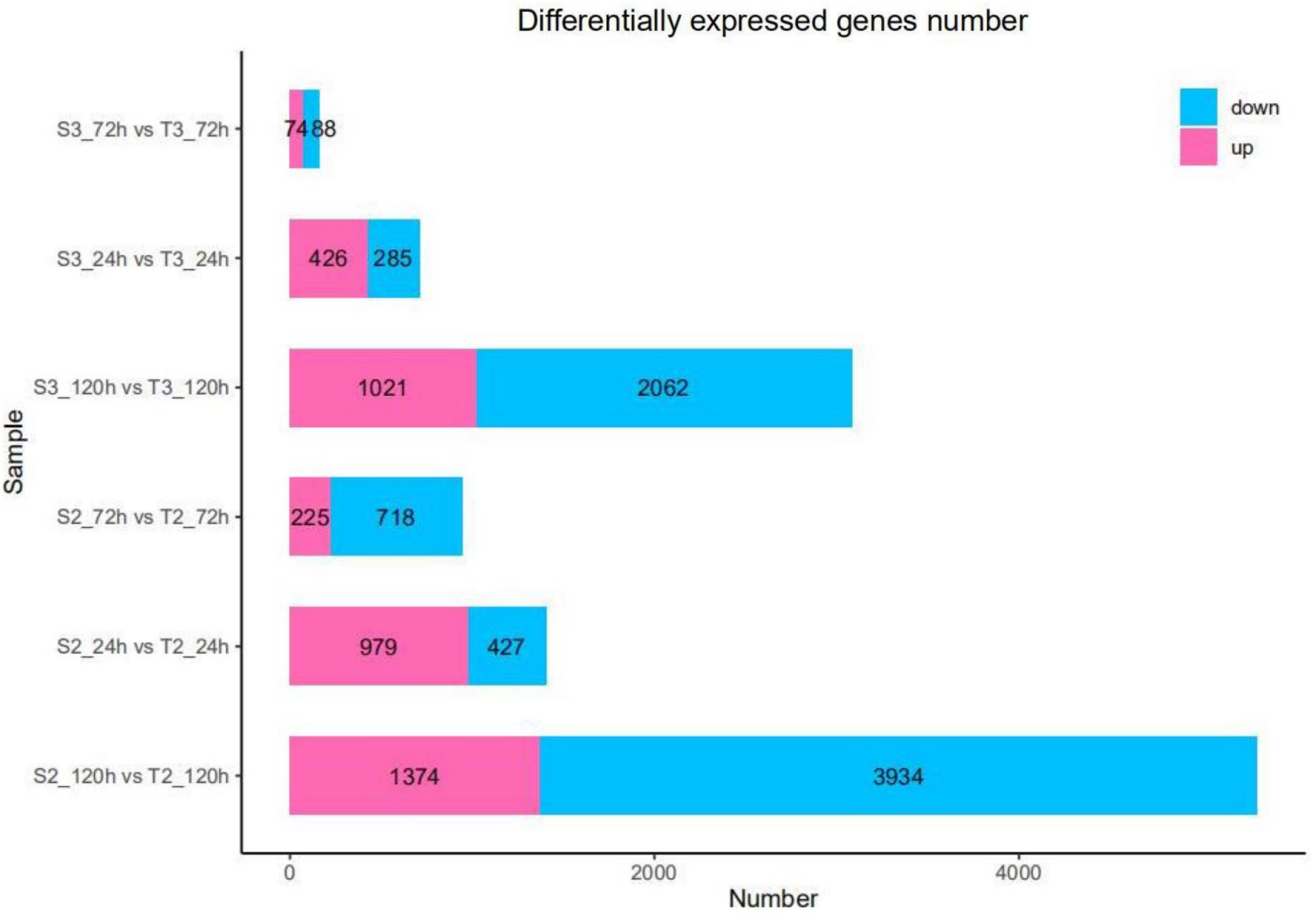
DEGs statistics at different stages of Xinluzao-36 and Zhongzhimian-2 after *V. dahliae* fungus infection. The up-regulated DEGs are shown in red, while the down-regulated DEGs are shown in blue in the stacked bar graphs.

Differential expression analysis showed that 162 genes were differentially expressed between the S3-72H vs T3-72H groups (74 upregulated and 88 downregulated), 711 genes were differentially expressed between S3-24H vs T3-24H group (426 upregulated and 285 downregulated), 3083 genes were differentially expressed between S3-120H vs T3-120H group (1021 upregulated and 2062 downregulated), 943 genes were differentially expressed between S2-72H vs T2-72H group (225 upregulated and 718 downregulated), 1406 genes were differentially expressed between S2-24H vs T2-24H group (979 upregulated and 427 downregulated) and 5308 genes were differentially expressed between S2-120H vs T2-120H group (1374 upregulated and 3034 downregulated) (Fig. 2). Notably, maximum number of differentially expressed genes was observed at 120 h post *V. dahliae* fungus infection.

**Figure 3:**
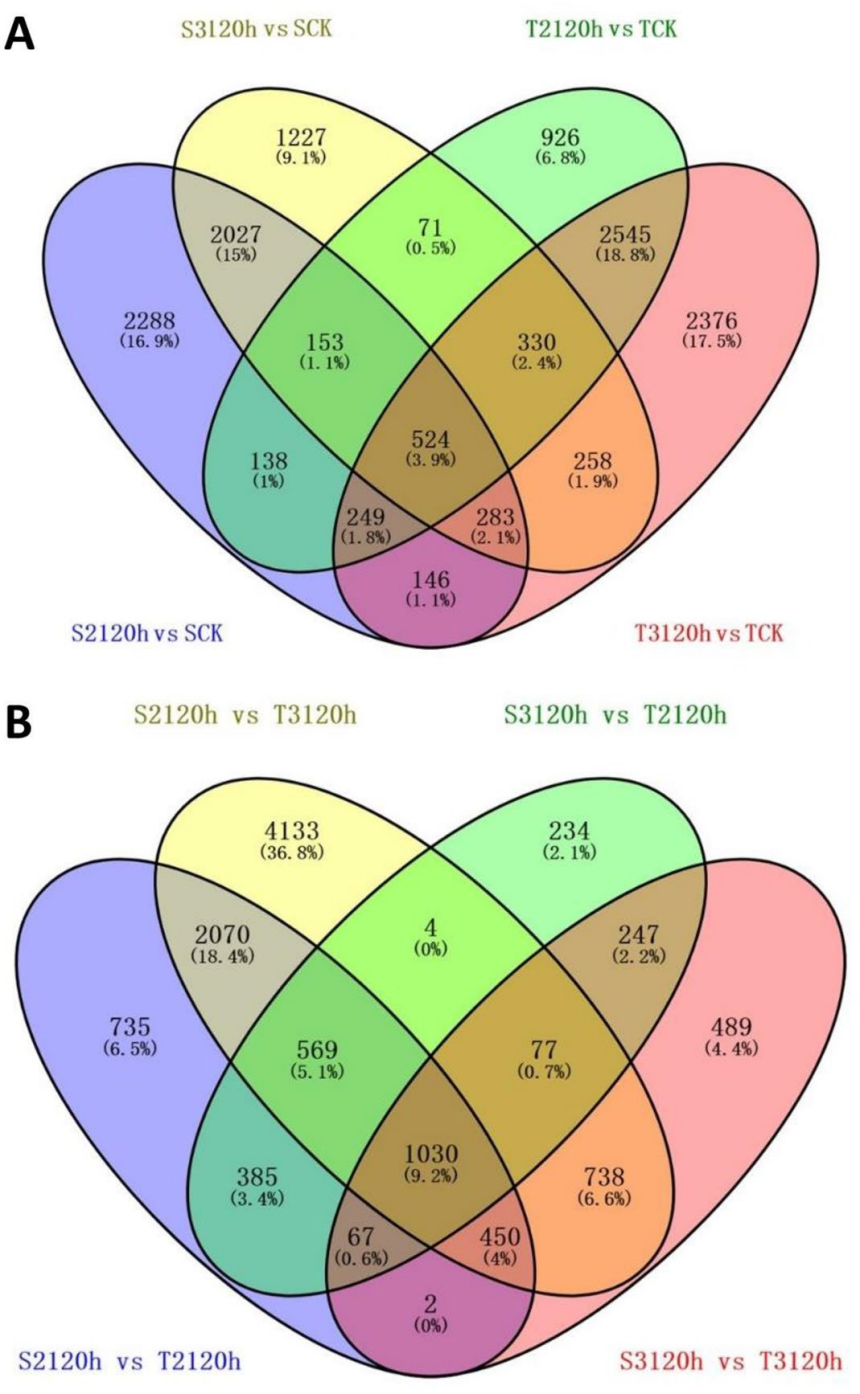
Venn diagram showing overlap of genes of *G. hirsutum* in response to *V. dahliae*. (**A**) Venn diagram showing overlap of up-regulated genes between the treatment groups and their respective control groups. (**B**) Overlap genes between the two treatment groups of in response to *V. dahliae*.

In the Xinluzao-36 and Zhongzhimian-2 response to *V. dahliae* fungus infection, 13,541 genes were upregulated in at least one time-point when the treatment group was compared to the control group (Fig. 3A). In the S2_120h vs SCK, 5808 genes were differentially expressed while 4,936 were differentially expressed in the T2_120h vs TCK group. Equally, 4873 were differentially expressed in S3_120h vs SCK, and 6711 were differentially expressed in the T3_120h vs TCK group. Comparison between the treatment groups, 5308 were differentially expressed in the S2_120h vs T2_120h group while 3100 were differentially expressed in the S3_120h vs T3_120h group. Equally, 9071 were differentially expressed in S2_120h vs T3_120h, and 2613 were differentially expressed in the S3_120h vs T2_120h group (Fig. 3B).

### Functional classification and enrichment analysis of DEGs

**Figure 4:**
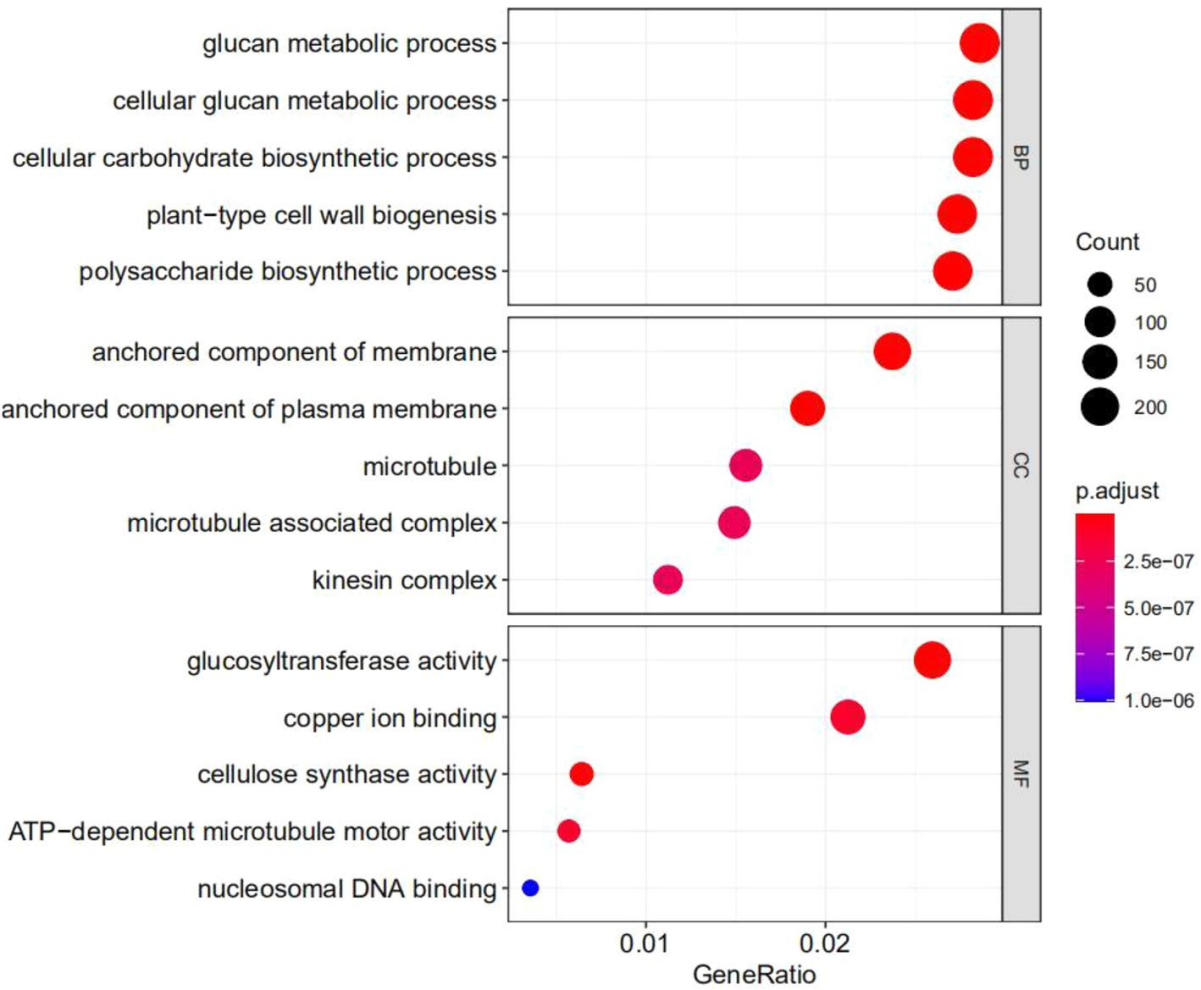
Gene Ontology (GO) term enrichment analysis. An FDR of < 0.05 was used to pick significantly enriched GO terms.

In comparisons of libraries made for the various time points, GO enrichment approaches were used to identify the putative roles of DEGs (Fig. 4). GO enriched categories of the DEGs in biological process groups were primarily linked in glucan metabolism process, cellular carbohydrate biosynthesis process, plant-type cell wall Biogenesis and polysaccharide biosynthesis. The GO enriched categories of the DEGs in cellular component were mostly linked to the anchored component of membrane, anchored component of plasma membrane, microtubule, microtubule associated complex and kinesin complex. GO enriched categories of the DEGs in molecular function were mostly linked to glucosyltransferase activity, copper iron binding, cellulose synthase activity and ATP-dependent microtubule motor activity (Fig. 4).

**Figure 5:**
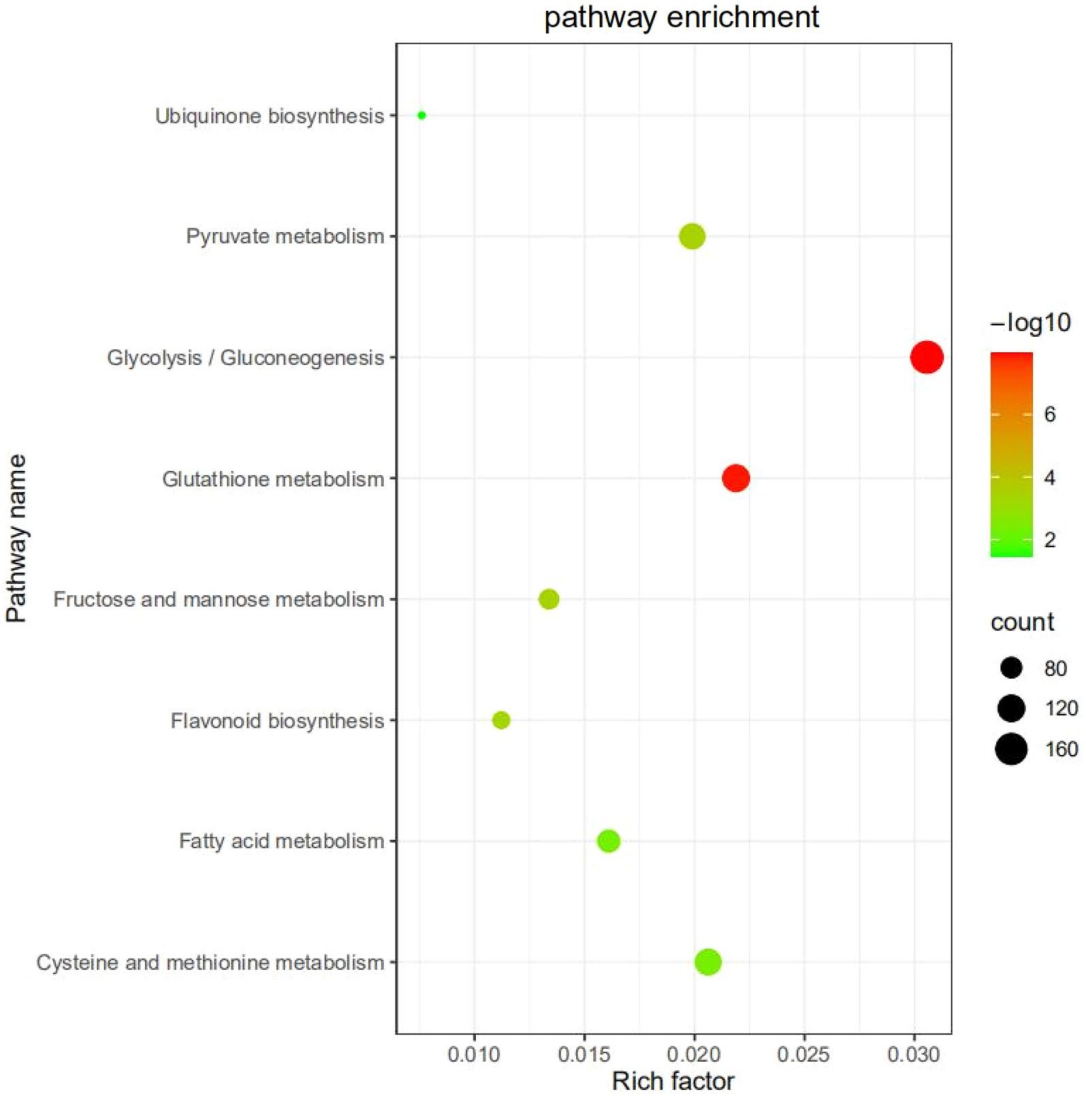
Analysis of KEGG pathways enrichment. Each circle in the diagram represents a KEGG metabolic pathway, and the size of the circle corresponds to the number of genes enriched in that pathway. The importance of DEG enrichment in a pathway is represented by the − log_10_ scale (qvalue).

We also conducted enrichment analysis by mapping sequences to KEGG database groups in order to examine the roles of the differentially expressed transcripts. KEGG annotated DEG’s were allocated to various classes, mainly related to biosynthesis of other secondary metabolites, signal transduction and carbohydrate metabolism. KEGG enrichment analyses as well showed DEGs were considerably enriched in pathways such as in ubiquitin biosynthesis, pyruvate metabolism, gluconeogenesis, glutathione metabolism, fructose and mannose metabolism, Flavonoid biosynthesis, fatty acid metabolism, cysteine and methionine metabolism (Fig. 5).

### Weighted gene expression clustering analysis

**Figure 6:**
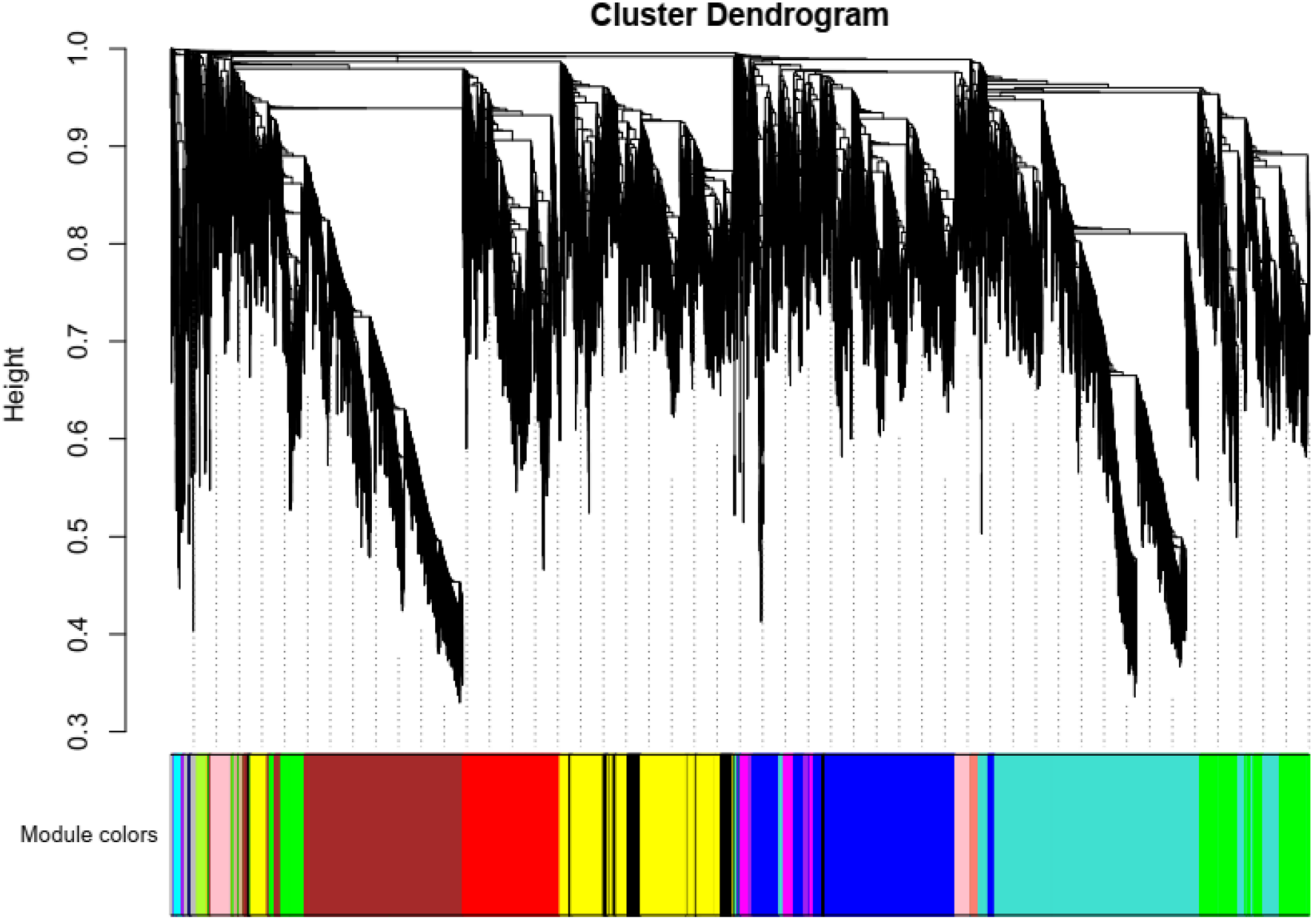
Weighted gene correlation network analysis (WGCNA). Hierarchical clustering of the topological overlap matrix for *V. dahliae* expression data.

A popular approach to comparative genomics is to identify and annotate DEG sets for the purpose of mining the key genes. Therefore, WGCNA was conducted to further examine the basic molecular pathways and key genes affecting *V. dahliae* growth within the two cotton varieties. According to similar expression and similar functions, we finally selected all the differential genes (13,541) for 120 h for the WGCNA analysis. The soft power was set at 15 since this was the lowest power required to attain nearly scale-free topology (R2 = 0.865), and the number of co-expression modules was 16 modules in total (Fig. 6).

As per the correlation results between the modules and the two sample treatments in each phase, the pink module and S sample that are significantly positively correlated with T sample at 120 h and negatively correlated with S sample at120h were selected. The 120 h significant positive correlation of the T sample and the large negative correlation of the T sample 120 h brown module are used to construct the interactive network and hub screening of genes.

**Figure 7:**
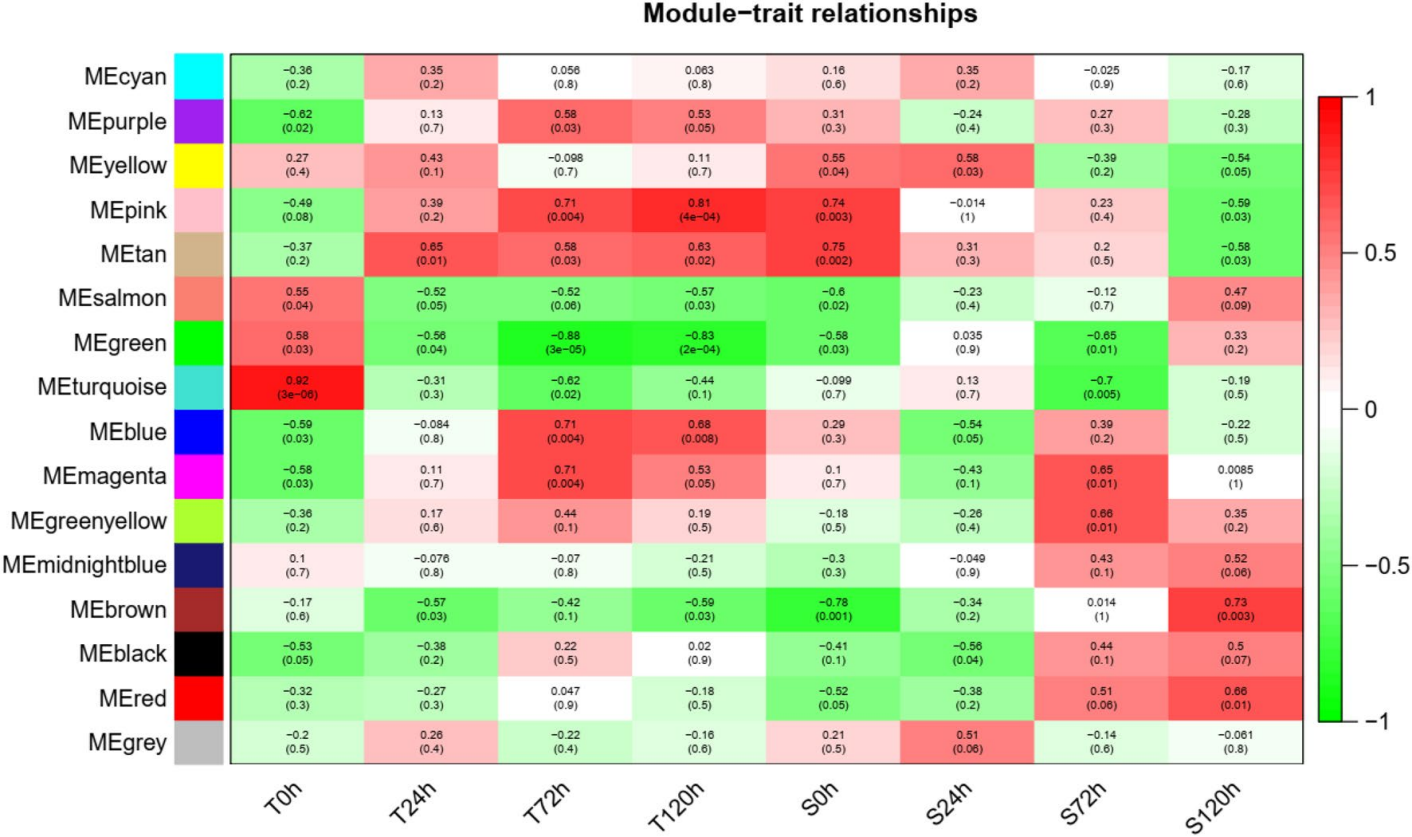
Module-trait correlation of *G. hirsutum* in response to *V. dahliae* fungus infection at specific time points.

From the module trait correlation analysis, 3 modules had an analogous down-regulation in four time points that include T24 h, T72 h, and T120 h, in the MECyan, MEpurple, MEyellow and MEpink modules. Whereas MEbrown and MEred were upregulated at S120 hours post inoculation (hpi), and Mgrey encompassed upregulated genes that were at both S0 hpi and S24 hpi. Equally, MEyellow contained genes that were both upregulated in the T0hpi up to the T120hpi. Thus, each of these modules recognizes a gene set of *G. hirsutum* in response to *V. dahliae* fungus infection at specific time points (Fig. 7).

### Networks displaying relationships among genes within co-expressed modules

**Figure 8:**
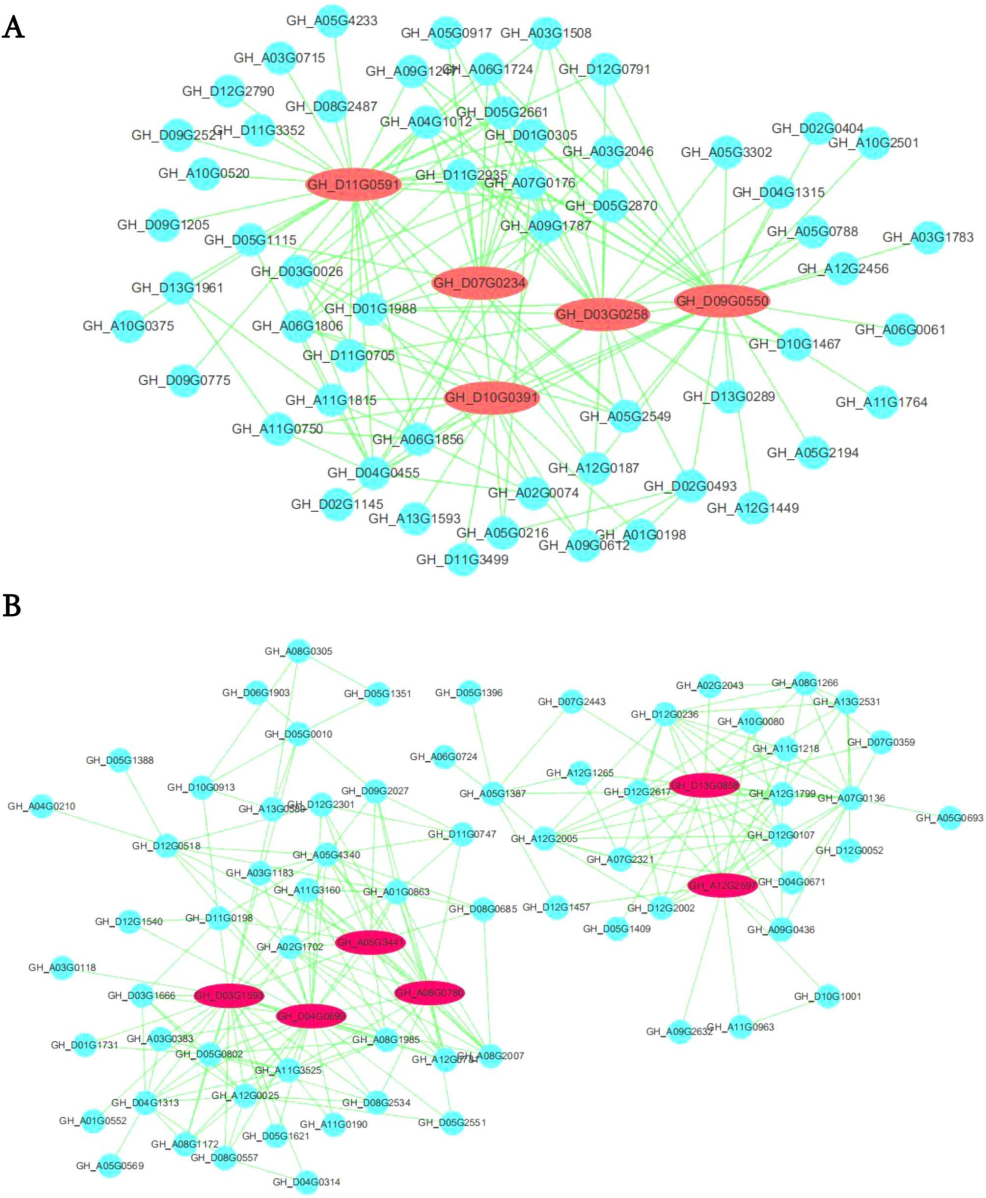
Co-expression network analysis of brown (**A**) and pink (**B**).

With the goal of identifying main hub genes, we built a network of the identified co-expressed modules. In this study, we identified 11 hub genes that encode for different proteins associated with lignin and immune response, Auxin response factor, cell wall and vascular development, microtubule, Ascorbate transporter, Serine/threonine kinase and Immunity and drought (Fig. 8, Table 2).

**Table 2:** The hub genes detected in the two WGCNA modules.

Modules	Gene_id	Arabidopsis orthologs	Comparison rate	Predicted functions
Brown	GH_D07G0234	AT2G26180	61.33	Microtubule related
	GH_D09G0550	AT5G51600	65.22	Microtubule related
	GH_D10G0391	AT2G34190	83.85	Ascorbate transporter
	GH_D11G0591	AT5G10010	67.73	Serine/threonine kinase
	GH_D03G0258	AT5G67270	65.99	B1 plays a role in microtubules during mitosis and early cytokinesis
Pink	GH_A05G3441	AT3G55830	65.34	Related to lignin and immune response
	GH_A08G0780	AT5G62000	51.63	Auxin response factor
	GH_A12G2597	AT5G65310	55.56	Related to cell wall and vascular development
	GH_D13G0858	AT1G13740	70.45	Immunity and drought related
	GH_D03G1593	AT1G07110	77.6	Encoding the bifunctional enzyme fructose 6-phosphate 2-kinase/fructose-2,6 bisphosphatase
	GH_D04G0699	AT3G55830	65.64	Related to Arabidopsis immunity

### qPCR validation

**Figure 9:**
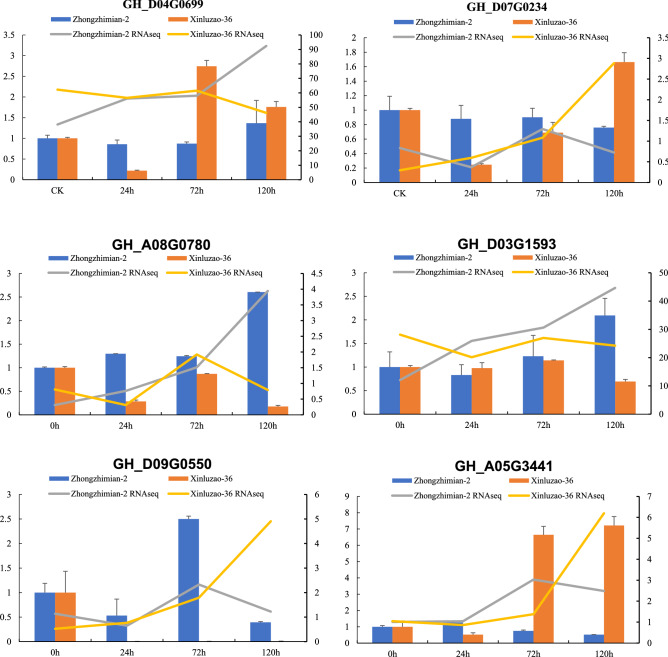
Real time-PCR validation of DE transcripts.

A set of differentially genes were randomly selected for detection and subsequent validation via qPCR, Fig. 9.

## Discussion

Generally, Verticillium wilt restrains water and nutrient movement within the cotton vascular system, and considerably limits the crop yields. Equally, the photosynthetic rate and other related physiological traits in cotton genotypes are severely influenced by the Verticillium wilt^38^. Cotton genotypes display diverse reactions and resistance mechanisms to Verticillium wilt infection^39^. Mechanisms for *V. dahliae* Resistance may involve modifications to the transcription factors, extracellular enzymes, cell wall, ethylene-associated signal transduction pathways, jasmonic acid pattern recognition receptors or salicylic acid^40^.

To aid infection and efficient colonization, pathogenic fungi in plants can develop a variety of cell wall-degrading enzymes^41,42^, including pectinase, hemicellulase, cellulase, etc. Hydrolytic enzymes, especially cellulases and pectinases, have long been thought to play a key role in the expression of *V. dahliae* disease symptoms and pathogenesis^43,44^. The cell wall-degrading enzymes are virulence factors, for instance xyloglucan-specific endoglucanase^45^, fungal endopolygalacturonases^46^, as well operate as pathogen-associated molecular patterns (PAMPs). In particular, enzymes linked in cell wall degradation comprise non-catalytic domains, which are usually related to the fungal carbohydrate hydrolases, considered to be activators of the PAMP-triggered immunity (PTI) response in the oomycetes^47,48^. In *V. dahliae*, two Glycoside hydrolase 12 (GH12) proteins; VdEG1 and VdEG3 acted as PAMPs that triggered cell death and PTI independent of their enzyme action in *Nicotiana benthamiana*. Despite the fact that cell wall-degrading enzymes have also been linked to fungus pathogenicity, direct molecular evidence was insufficient. Herein, some of the enriched GO classifications are significantly related to the composition of the cell wall turn off. They include cellular carbohydrate biosynthesis process, plant-type cell wall Biogenesis and polysaccharide biosynthesis. Considering the main component of the cell wall is sugar, this observed pattern of enriched pathways may form part of the cell wall defense mechanism against pathogens at the initial stage of *V. dahliae* infection.

Phenylpropanoids, which naturally comprise of flavonoids and lignin, typically perform vital functions that are associated with biotic stress response in plant by both availing material units for physical barriers as well as synthesizing various antibiotic compounds^49,50^. Further, lignin has been identified as a contributing factor in the resistance mechanisms of cotton to diseases^51^. Specifically, phenylpropane synthesis is associated with defense mechanisms in cotton^52^, whereas flavonoids are linked to cushion considerable stress-induced changes in the reactive oxygen species (ROS) homeostasis and modulation of the ROS-signaling pathway^53^. ROS are vital in defense signaling mechanisms^54^ as well as controlling the programmed cell death by means of formation of the HR^55^. In this study, flavonoid biosynthesis was highly upregulated in the *V. dahliae* infected cotton varieties. In line with previously conducted studies that have described the significance of lignin pathway in lignification and reinforcement of cell walls as a vital processes in plant responses to fungal infection^51,56^, we identified significant lignin pathway enrichment that was more established in the disease resistant variety more than in the disease susceptible *V. dahliae* variety. Although there is no much documented literature on the glutathione metabolism pathways in the transcriptome studies conducted on cotton and treated with *V. dahliae*, much has been reported in tomatoes elsewhere. Indeed, Sulfur is an effective macronutrient in plants as it can help them fight disease by forming sulfur-containing defense compounds (SDCs) including glutathione-S-transferase, glutathione, phytochelatins, as well as other sulfur-containing proteins^57^, and increased sulfur availability aids in boosting plant disease resistance to pathogens^58–61^. In accordance to previously conducted studies, SDC aggregation is quickly increased in pathogen-resistant cultivars compared to the susceptible cultivars. Similarly, other numerous studies have shown the importance of sulfur in disease resistance in plants^62–64^. In tomatoes, expression levels of genes linked to sulfate uptake that has been observed as well as the integration and the SDCs formation were all up-regulated, thus indicating that sulfur-enhanced defense may be playing a significant part in improved resistance of tomatoes to *V. dahliae*, as depicted by Rausch and Wachter^57,65^. Further, the increased GSH content together with other up-regulated genes expression associated with sulfur metabolism, like those that encode for glutathione-S-transferase, cysteine synthase and peptide methionine sulfoxide reductase, possibly play a part in enhancing the resistance against *V. dahliae* in tomatoes^65^. Intriguingly, Glutathione metabolism was upregulated in the KEGG enrichment pathway. Thus, it may be implicated in the resistance mechanisms of *V. dahliae* infection in cotton varieties.

WGCNA split the core DEG’s into two modules, each providing a distinct metabolism pathway in relation to *V. dahliae* infection. From the recognized hub genes, co-expression network of upregulated hub genes comprised of Ascorbate transporter, Serine/threonine kinase, Auxin response factor, and Immunity and drought related genes. In accordance with GO term analysis, these hub genes were involved in the transport and in the immune response mechanisms. Plants utilize a wide variety of chemicals to defend them from pathogens. These chemical compounds may build up in the affected tissue at high levels^66^. Proteins encompassed in long distance transportation are important for the transfer of these protective compounds to other tissues and for the survival of plants under a viral attack^67^. The differentially expressed transporters recognized herein may have a part in transportation of secondary metabolites as well as the translocation of defense associated compounds that stimulate other mechanisms intricate in defense responses of plants when under a viral attack. Serine/threonine protein kinase (STK) performs a significant part in plant stress-signaling transduction pathway through phosphorylation^68^. Serine/threonin protein kinase (STK) is a major defensive signal transduction protein. The primary function of STK in plant–microbe systems is to sense and relay environmental signals from a pathogen^69^. Equally, phytohormone auxin also plays the key role in controlling many different characteristics of plant development, including lateral root initiation, elongation of the shoot, embryogenesis, vascular growth, tropical development and architecture of flowers and tissues^70,71^. Auxin response factors (ARFs), which are essential apparatuses of the auxin signal pathways, control the expression of auxin-responsive genes by binding directly in their promoter regions to the auxin-responsive variable^72^.

## Supplementary Information

Supplementary Information.

## Data Availability

This study data can be accessed through the NCBI Sequence Read Archive under Bio project ID number PRJNA745369, with SRA numbers SRR15144248, SRR15144249, SRR15144250, SRR15144251, SRR15144252, SRR15144253, SRR15144254, SRR15144255, SRR15144256, SRR15144257, SRR15144258, SRR15144259, SRR15144260, SRR15144261, SRR15144262, SRR15144263, SRR15144264, SRR15144265, SRR15144266, SRR15144267, SRR15144268, SRR15144269, SRR15144270, SRR15144271, SRR15144272, SRR15144273, SRR15144274, SRR15144275, SRR15144276, SRR15144277, SRR15144278, SRR15144279, SRR15144280, SRR15144281, SRR15144282, SRR15144283, SRR15144284, SRR15144285, SRR15144286, SRR15144287, SRR15144288 and SRR15144289.
